# The roles of serum vitamin D and tobacco smoke exposure in insomnia: a cross-sectional study of adults in the United States

**DOI:** 10.3389/fnut.2023.1285494

**Published:** 2023-12-18

**Authors:** Tianci Gao, Mengxing Hou, Qianfei Wang, Dong Liu, Fenqiao Chen, Yueyi Xing, Jianqiang Mei

**Affiliations:** ^1^Graduate School, Hebei University of Chinese Medicine, Shijiazhuang, Hebei, China; ^2^Department of Emergency, First Affiliated Hospital, Hebei University of Chinese Medicine, Shijiazhuang, Hebei, China; ^3^Department of Traditional Chinese Medicine, Hebei General Hospital, Shijiazhuang, Hebei, China; ^4^School of Basic Medicine, Hebei University of Chinese Medicine, Shijiazhuang, Hebei, China

**Keywords:** cotinine, vitamin D, insomnia, regulating effect, NHANES

## Abstract

**Aim:**

Tobacco smoke exposure and vitamin D (VD) status were both associated with insomnia. However, the combined effect of smoking and VD on insomnia has not been discussed. This study aimed to explore the role of VD in the association between tobacco smoke exposure and insomnia.

**Methods:**

Data on adults were extracted from the National Health and Nutrition Examination Surveys (NHANES) database in 2005–2008 for this cross-sectional study. Weighted univariate and multivariate logistic regression analyses were used to explore the associations between serum cotinine, serum VD, and insomnia. A surface diagram was drawn to reflect the effect of VD on the association between serum cotinine and insomnia. In addition, the potential regulating effect of VD in subgroups of smoking status was also performed. The evaluation index was odds ratios (ORs) with 95% confidence intervals (CIs).

**Results:**

Among the eligible participants, 1,766 had insomnia. After adjusting for covariates, we found that elevated serum cotinine levels were associated with higher odds of insomnia [OR = 1.55, 95% CI: (1.22, 1.97)]. However, the relationship between serum VD level and insomnia was not significant (*P* = 0.553). Higher serum cotinine levels were also associated with higher odds of insomnia [OR = 1.52, 95% CI: (1.17, 1.98)] when serum VD level was <75 nmol/L; however, this relationship became non-significant when serum VD concentration was elevated (*P* = 0.088). Additionally, the potential regulating effect of VD was also found in adults who were not smoking.

**Conclusion:**

VD may play a potential regulative role in the association between tobacco smoke exposure and insomnia. Further studies are needed to clarify the causal relationships between VD, tobacco smoke exposure, and insomnia.

## Introduction

Insomnia is a highly prevalent sleep problem in clinical settings, and up to 50% of primary care patients are plagued by it (1). The primary characteristics of insomnia are prolonged sleep latency and difficulty maintaining sleep, which are accompanied by symptoms such as irritability or fatigue when awake (2). Insomnia is a risk factor for impaired functioning, the development of other physical and mental disorders, and increased healthcare costs (2). Therefore, accurate identification of the influencing factors for insomnia and finding appropriate interventions are of great significance to reducing the burden of the disease.

The etiology and pathophysiology of insomnia involve genetic, environmental, behavioral, and physiological factors, which ultimately lead to excessive arousal (3, 4). Tobacco smoke is a common environmental exposure factor and has become a serious public health problem (5). As the main acting substance in tobacco, nicotine may influence the release of neurotransmitters such as dopamine and thus affect sleep (6). Furthermore, nicotine may stimulate cholinergic neurons in the basal forebrain and cause physiological arousal (7). A recent systematic review and meta-analysis reported that second-hand smoke exposure was associated with short sleep duration and poor sleep quality (7). Another study used polysomnography to evaluate sleep in smokers and non-smokers, which also showed that smokers had a number of insomnia-like sleep impairments compared to non-smokers (8).

A growing body of research has revealed that vitamin D (VD) plays an important role in regulating brain function (9, 10). Animal experiments and immunohistochemistry of the human brain have found that nuclear receptors for VD are widely present in many brain regions, such as the hypothalamus, raphe nucleus, and substantia nigra, which are thought to play a key role in sleep regulation (11–13). In addition, VD is involved in the production of melatonin and the dopaminergic system, which is related to the regulation of human circadian rhythms and sleep (14, 15). A population-based study has found that people with VD deficiency have a significantly increased risk of sleep disorders (16). Patients with insomnia were found to have significantly lower serum VD levels than healthy people, and those with insufficient responses to treatment in patients with insomnia had lower serum VD levels (13).

Previous studies have found that VD and tobacco exposure have a combined effect on a variety of diseases, including children's lung function, spontaneous abortion, and hypertension (17–19). A recent study on depression and lifestyle showed that both inadequate intake of VD and smoking can increase the risk of depression, and insomnia is involved in the pathogenesis of depression (20). However, the interaction effect of VD and smoking on insomnia, as well as the underlying mechanisms, are unknown. This study aimed to explore the combined effect of serum VD concentration and tobacco smoke exposure on insomnia, as well as whether VD can reduce the risk of insomnia associated with tobacco smoke exposure. We hope that this study may provide some references for the prevention and treatment of insomnia in clinical practice.

## Methods

### Study design and population

The demographic and clinical data of participants in this retrospective cohort study were extracted from the National Health and Nutrition Examination Surveys (NHANES) database in 2005–2008. The NHANES survey is conducted by both the Centers for Disease Control and Prevention (CDC) and the National Center for Health Statistics (NCHS) with the aim of assessing the nutritional and health status of the non-institutionalized population in the United States. This database includes a complex, multistage stratified probability sample based on selected counties, blocks, households, and persons within households. NCHS-trained professionals conducted interviews in the participants' homes, and extensive physical examinations were conducted at mobile exam centers (MECs). More details are available for online access: https://wwwn.cdc.gov/nchs/nhanes/Default.aspx.

The inclusion criteria were (1) age >20 years old and (2) insomnia assessment information. A total of 10,890 adults were initially included. The exclusion criteria were (1) missing information on tobacco smoke exposure, serum VD concentration, total energy intake, caffeine, Healthy Eating Index-2015 (HEI-2015), pregnancy, cancer, or taking tranquilizers or sleep medication and (2) missing information on body mass index (BMI), poverty-income ratio (PIR), education level, drinking, smoking, depression, or C-reactive protein (CRP). Finally, 6,312 individuals were eligible. The NHANES was approved by the institutional review board (IRB) of NCHS. The study data were de-identified, and all the participants provided informed consent. No ethical approval of our agency's IRB was required since the NHANES is publicly available.

### Examination of serum vitamin D concentration

The blood samples of participants were collected through physical examinations by trained professionals at MECs, divided into aliquots, and shipped to multiple laboratories for analysis. NHANES measured serum 25-hydroxyvitamin D [25(OH)D] concentration to reflect VD status using a standardized liquid chromatography-tandem mass spectrometry (LC-MS/MS) method. In NHANES, the serum VD examination was performed within two 6-month time periods, including 1 November to 30 April and 1 May to 31 October. Additionally, the examination time during the day included the morning, afternoon, and evening. In this study, we divided the serum VD concentration into two levels according to a previous study, that is, ≥75 nmol/L represented an adequate intake of VD, while < 75 nmol/L represented an inadequate intake of VD (21).

### Assessment of tobacco smoke exposure

Tobacco smoke exposure status was reflected by serum cotinine concentration, which is a biomarker of exposure to combustible and non-combustible tobacco products. Serum cotinine was assessed by NHANES, with samples analyzed using isotope-diluted high-performance liquid chromatography (22). Following recommendations and prior NHANES research (23, 24), we used optimal cut points to distinguish between no/minimal exposure: cotinine < 0.05 ng/mL, low exposure: cotinine of 0.05–2.99 ng/mL, and high exposure: cotinine ≥3.00 ng/mL.

### Measurement of insomnia

The diagnosis of insomnia was according to the NHANES sleep disorders questionnaires (SLQs) with three questions, including the frequency of difficulty falling asleep (SLQ080), prolonged nocturnal awakening (SLQ090), and undesired early morning awakening (SLQ100) over the past month. Individuals were recognized as patients with insomnia if they responded affirmatively to experiencing at least one of the above symptoms at least five times in the past month (25).

### Variables collection

We collected potential covariates including age, sex, race, educational level, PIR, work status, work shift, BMI, physical activity, drinking, smoking, hypertension, diabetes mellitus (DM), dyslipidemia, depression, obstructive sleep apnea (OSA), CRP, total energy intake, caffeine intake, HEI-2015, and the collection season and time of serum VD.

During the NHANES household interview, smoking status, the pattern of alcohol consumption, work status, and work shift were captured using questionnaires. During the face-to-face MEC interview, depressive symptoms over the last 2 weeks were assessed. The depressive symptoms included anhedonia, depressed mood, sleep disturbance, fatigue, appetite changes, low self-esteem, concentration problems, psychomotor disturbances, and suicidal ideation. Total scores range from 0 to 27, with scores ≥10 representing clinically significant depressive symptoms. Depression was diagnosed by the above scores as well as taking antidepressants. Patients with total cholesterol (TC) ≥200 mg/dL (5.2 mmol/L) or triglycerides (TGs) ≥150 mg/dL (1.7 mmol/L) or low-density lipoprotein cholesterol (LDL-C) ≥130 mg/dL (3.4 mmol/L) or HDL-C ≤40 mg/dL (1.0 mmol/L) or self-reported hypercholesterolemia or receiving lipid-lowering therapy were identified as dyslipidemia. DM was defined according to a self-reported diagnosis, the use of oral hypoglycemic agents or insulin, glycosylated hemoglobin (HbAlc) ≥6.5%, a plasma glucose level of ≥200 mg/dL at 2 h after the oral glucose tolerance test (OGTT), or a fasting glucose level of ≥126 mg/dL. OSA was diagnosed through a positive answer to the following questions: doctor diagnosed sleep apnea (SLQ070A), “How often do you snore?” (SLQ030), with the answer of ≥3 nights/week, “How often do you snort/stop breathing?” (SLQ040) with the answer of ≥3 nights/week, feeling excessively sleepy during the day, with the answer of 16–30 times per month despite sleeping ~7 or more hours per night on weekdays or work nights (SLQ120).

Total energy intake was calculated by “Total nutrient intakes” and “Total dietary supplements” from 24-h dietary recalls of NHANES. BMI was divided into two levels according to the criteria of the WHO: underweight/normal (BMI ≤ 25 kg/m^2^) and overweight/obesity (BMI > 25 kg/m^2^). Physical activity was converted to metabolic equivalent (MET), which was calculated according to the physical activity questionnaire (PAQ) in NHANES. Energy expenditure (MET·min) = recommended MET × exercise time of corresponding activity (min).

### Statistical analysis

The normal distribution data were described using mean ± standard error (mean ± SE) and a *t*-test for comparison. Enumeration data were expressed as frequency and constituent ratio [N (%)] and chi-square test (χ^2^) for groups' comparison. The NHANES special weight “2-year sample weights (WTMEC2YR)” was used for combined analyses of the two 2-year cycle's data (2005–2008), and more details are available on the website: https://wwwn.cdc.gov/nchs/nhanes/tutorials/Weighting.aspx.

Weighted univariate logistic regression analysis was used for covariate screening. We used both univariate and multivariate logistic regression analyses to explore the association between serum VD, serum cotinine, and insomnia, respectively. Model 1 was the crude model. Model 2 was adjusted for age, sex, race, PIR, smoking, hypertension, DM, dyslipidemia, depression, OSA, CRP, total energy intake, work status, work shift, and VD collection season. We drew a surface diagram to reflect the effect of VD on the association between serum cotinine concentration and insomnia. In addition, we explored the potential regulating effect of VD on the relationship between cotinine and insomnia in smoking subgroups. The evaluation index was odds ratios (ORs) with 95% confidence intervals (CIs). A two-sided *P*-value of < 0.05 is considered statistically significant.

Statistical analyses were performed using R (version 4.2.0, Institute for Statistics and Mathematics, Vienna, Austria) and SAS 9.4 (SAS Institute, Cary, NC, USA). Missing data were deleted, and sensitivity analysis of participants' characteristics before and after the deletion of missing data was performed (Supplementary Table 1).

## Results

### Characteristics of the study population

**Figure 1:**
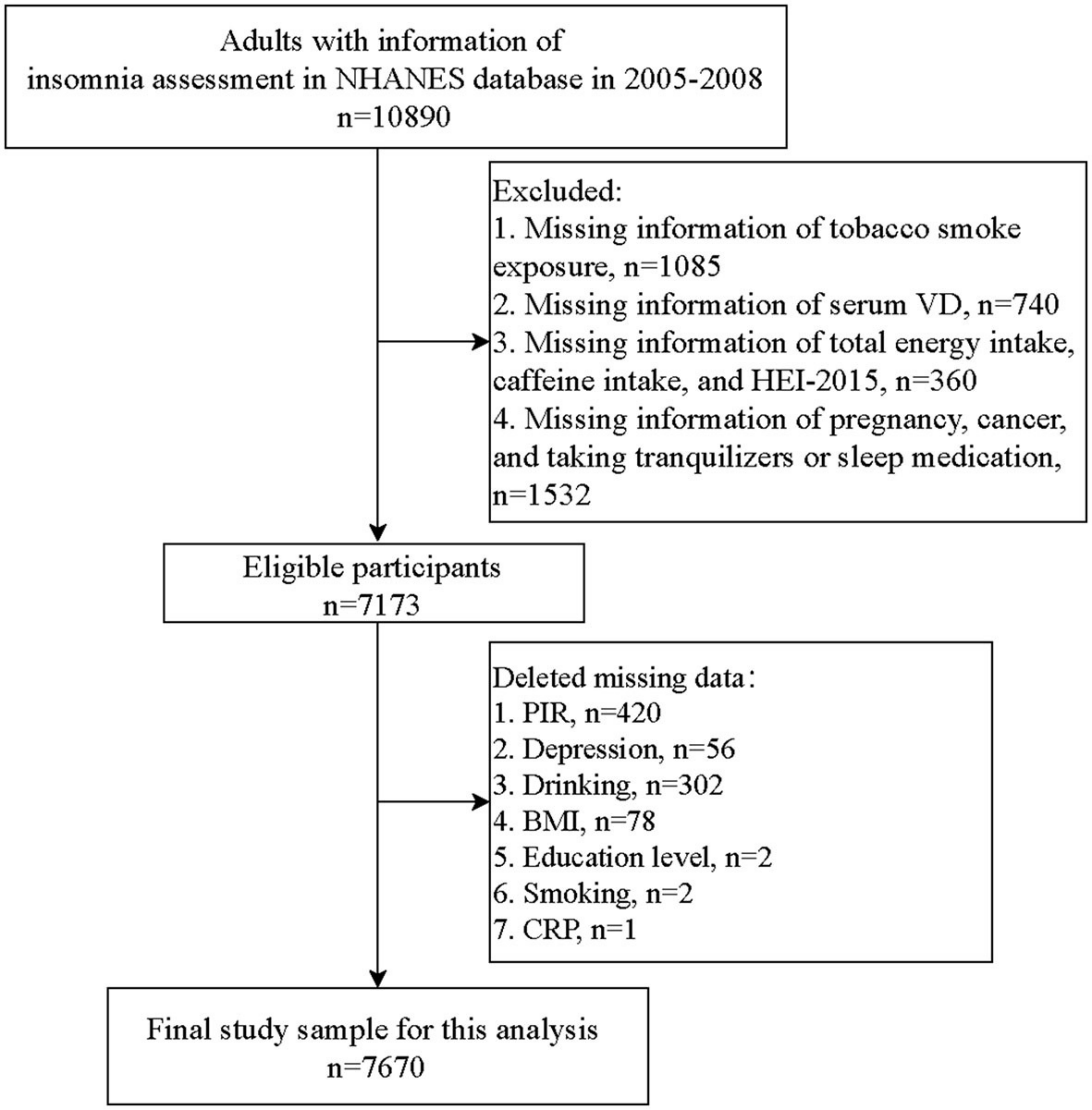
Flowchart of participant screening.

Figure 1 shows the flowchart of the participants' screening. There were 10,890 adults with information about insomnia assessments in the NHANES database. Individuals without information about tobacco smoke exposure (*n* = 1,085), serum VD concentration (*n* = 740), total energy intake, caffeine intake, or HEI-2015 (*n* = 360), pregnancy, cancer, or taking tranquilizers or sleep medication (*n* = 1,532) were excluded. Missing information on variables including PIR (*n* = 420), depression (*n* = 56), drinking (*n* = 302), BMI (*n* = 78), education level (*n* = 2), smoking (*n* = 2), and CRP (*n* = 1) were deleted. Finally, 6,312 people were eligible.

The characteristics of eligible participants and covariates associated with insomnia are shown in Table 1. A total of 1,766 (27.98%) had insomnia. The average age of the study population was 45 years old, and 3,278 (50.80%) were male. Among the patients with insomnia, serum cotinine levels <0.05 ng/mL accounted for 39.32%, followed by ≥3 ng/mL (35.60%). Most people had serum VD levels of <75 nmol/L in the non-insomnia group or insomnia group. In addition, age, sex, race, PIR, work status, work shift, smoking, hypertension, DM, dyslipidemia, depression, OSA, CRP, total energy intake, and VD collection season were all significantly associated with insomnia and were included in the adjustment of multivariate models (all *P* < 0.05).

**Table 1:** Characteristics of eligible participants.

**Variables**	**Total (*n* = 6,312)**	**Non-insomnia (*n* = 4,546)**	**Insomnia (*n* = 1,766)**	**Covariates screening**	
				**OR (95% CI)**	***P***
Age, years, mean ± SE	45.04 (0.45)	44.61 (0.45)	46.08 (0.61)	1.01 (1.00–1.01)	0.005
**Sex**, ***n*** **(%)**					
Male	3,278 (50.80)	2,479 (53.81)	799 (43.37)	Ref	
Female	3,034 (49.20)	2,067 (46.19)	967 (56.63)	1.52 (1.35–1.72)	< 0.001
**Race**, ***n*** **(%)**					
Mexican American	1,234 (8.42)	966 (9.58)	268 (5.56)	Ref	
Other hispanic	454 (4.10)	323 (4.05)	131 (4.22)	1.80 (1.33–2.43)	< 0.001
Non-hispanic white	3,033 (71.65)	2,072 (69.70)	961 (76.46)	1.89 (1.57–2.28)	< 0.001
Non-hispanic black	1,363 (10.90)	1,015 (11.32)	348 (9.84)	1.50 (1.22–1.84)	< 0.001
Other races	228 (4.93)	170 (5.35)	58 (3.92)	1.27 (0.88–1.82)	0.210
**Education level**, ***n*** **(%)**					
Less than 9th grade	728 (5.64)	518 (5.72)	210 (5.46)	Ref	
9–11th grade	1,043 (12.26)	730 (11.72)	313 (13.60)	1.22 (0.87–1.71)	0.268
High school grad/GED or equivalent	1,515 (24.40)	1,049 (23.11)	466 (27.59)	1.25 (0.93–1.69)	0.154
Some college or AA degree	1,745 (30.85)	1,271 (30.97)	474 (30.55)	1.03 (0.76–1.41)	0.835
College graduate or above	1,281 (26.85)	978 (28.48)	303 (22.80)	0.84 (0.61–1.16)	0.292
**PIR**, ***n*** **(%)**					
≤ 1	1,159 (11.85)	768 (10.84)	391 (14.35)	Ref	
>1	5,153 (88.15)	3,778 (89.16)	1,375 (85.65)	0.73 (0.60–0.87)	0.002
**Work status**, ***n*** **(%)**					
Working at a job or business	3,958 (71.26)	2,990 (74.23)	968 (63.90)	Ref	
Not working at a job or business	2,354 (28.74)	1,556 (25.77)	798 (36.10)	1.63 (1.41–1.88)	< 0.001
**Work shift**, ***n*** **(%)**					
A regular daytime schedule	2,958 (53.77)	2,253 (56.60)	705 (46.78)	Ref	
A regular evening/night shift	357 (5.86)	257 (5.70)	100 (6.23)	1.32 (1.05–1.67)	0.026
A rotating shift	297 (4.96)	221 (5.17)	76 (4.45)	1.04 (0.72–1.51)	0.835
Another schedule	345 (6.65)	258 (6.73)	87 (6.44)	1.16 (0.84–1.60)	0.386
Unknown	2,355 (28.76)	1,557 (25.80)	798 (36.10)	1.69 (1.46–1.96)	< 0.001
**BMI, kg/m**^2^, ***n*** **(%)**					
≤ 25	1,827 (31.69)	1,339 (32.25)	488 (30.30)	Ref	
>25	4,485 (68.31)	3,207 (67.75)	1,278 (69.70)	1.10 (0.98–1.23)	0.125
**Physical activity, MET min/week**, ***n*** **(%)**					
≤ 450	899 (15.34)	669 (15.93)	230 (13.89)	Ref	
>450	3,425 (58.19)	2,476 (58.20)	949 (58.18)	1.15 (0.94–1.40)	0.191
Unknown	1,988 (26.47)	1,401 (25.87)	587 (27.93)	1.24 (0.99–1.54)	0.066
**Drinking**, ***n*** **(%)**					
No	1,829 (24.28)	1,336 (24.55)	493 (23.63)	Ref	
Yes	4,483 (75.72)	3,210 (75.45)	1,273 (76.37)	1.05 (0.93–1.19)	0.450
**Smoking**, ***n*** **(%)**					
No	4,877 (76.28)	3,612 (78.30)	1,265 (71.29)	Ref	
Yes	1,435 (23.72)	934 (21.70)	501 (28.71)	1.45 (1.24–1.71)	< 0.001
**Hypertension**, ***n*** **(%)**					
No	2,949 (51.08)	2,228 (53.36)	721 (45.43)	Ref	
Yes	3,363 (48.92)	2,318 (46.64)	1,045 (54.57)	1.37 (1.14–1.66)	0.002
**DM**, ***n*** **(%)**					
No	4,877 (76.28)	3,612 (78.30)	1,265 (71.29)	Ref	
Yes	1,435 (23.72)	934 (21.70)	501 (28.71)	1.25 (1.07–1.45)	0.008
**Dyslipidemia**, ***n*** **(%)**					
No	1,660 (27.48)	1,236 (28.91)	424 (23.95)	Ref	
Yes	4,652 (72.52)	3,310 (71.09)	1,342 (76.05)	1.29 (1.10–1.51)	0.004
**Depression**, ***n*** **(%)**					
No	5,504 (86.50)	4,120 (89.59)	1,384 (78.87)	Ref	
Yes	808 (13.50)	426 (10.41)	382 (21.13)	2.31 (1.93–2.76)	< 0.001
**OSA**, ***n*** **(%)**					
No	3,117 (49.21)	2,319 (50.42)	798 (46.20)	Ref	
Yes	3,195 (50.79)	2,227 (49.58)	968 (53.80)	1.18 (1.06–1.33)	0.007
CRP, mg/dL, Mean ± SE	0.40 (0.01)	0.37 (0.01)	0.46 (0.03)	1.14 (1.06–1.22)	0.002
Total energy intake, Kcal, mean ± SE	2,251.75 (21.49)	2,273.64 (19.37)	2,197.62 (41.65)	1.00 (1.00–1.00)	0.069
Caffeine intake, mg, mean ± SE	197.25 (6.75)	193.67 (6.77)	206.10 (10.79)	1.00 (1.00–1.00)	0.211
HEI−2015, mean ± SE	49.52 (0.38)	49.72 (0.40)	49.02 (0.53)	1.00 (0.99–1.00)	0.159
**VD collection season**, ***n*** **(%)**					
November 1 to April 30	2,879 (39.48)	2,138 (40.62)	741 (36.68)	Ref	
May 1 to October 31	3,433 (60.52)	2,408 (59.38)	1,025 (63.32)	1.18 (1.02–1.36)	0.031
**Blood collection time**, ***n*** **(%)**					
Morning	3,051 (48.52)	2,201 (48.64)	850 (48.22)	Ref	
Afternoon	2,297 (34.01)	1,633 (33.26)	664 (35.85)	1.09 (0.96–1.23)	0.205
Evening	964 (17.47)	712 (18.10)	252 (15.93)	0.89 (0.71–1.11)	0.302
**Cotinine, ng/mL**, ***n*** **(%)**					
< 0.05	2,754 (43.39)	2,068 (45.03)	686 (39.32)	Ref	
0.05–2.99	1,743 (27.03)	1,288 (27.81)	455 (25.08)	1.03 (0.85–1.25)	0.745
≥3.00	1,815 (29.58)	1,190 (27.16)	625 (35.60)	1.50 (1.24–1.81)	< 0.001
**VD, nmol/L**, ***n*** **(%)**					
< 75	5,079 (73.81)	3,671 (73.99)	1,408 (73.37)	Ref	
≥75	1,233 (26.19)	875 (26.01)	358 (26.63)	1.03 (0.90–1.19)	0.657

OR, odds ratio; CI, confidence interval; M, mean; SE, standard error; PIR, poverty-income ratio; BMI, body mass index; MET, metabolic equivalent; DM, diabetes mellitus; CRP, C-reactive protein; HEI, Healthy Eating Index; OSA, obstructive sleep apnea; VD, vitamin D.

Statistics: t-test and chi-square test.

Covariate screening used the weighted univariate logistic regression analysis.

### Associations between vitamin, cotinine, and insomnia

We then explored the relationships between serum VD level and insomnia and serum cotinine level and insomnia, respectively (Table 2). After adjusting for covariates, we found that adults with elevated serum cotinine levels had higher odds of insomnia than those with serum cotinine levels <0.05 ng/mL [OR = 1.55, 95% CI: (1.22, 1.97)]. However, a higher serum VD level was not significantly associated with higher odds of insomnia (*P* = 0.553).

**Table 2:** Association between serum VD level and insomnia, serum cotinine level, and insomnia.

**Variables**		**Model 1**		**Model 2**	
		**OR (95% CI)**	***P***	**OR (95% CI)**	***P***
Cotinine, ng/mL	< 0.05	Ref		Ref	
	0.05–2.99	1.03 (0.85–1.25)	0.745	1.06 (0.87–1.29)	0.599
	≥3.00	1.50 (1.24–1.81)	< 0.001	1.55 (1.22–1.97)	0.006
VD, nmol/L	< 75	Ref		Ref	
	≥75	1.03 (0.90–1.19)	0.657	0.95 (0.80–1.12)	0.553

VD, vitamin D; OR, odds ratio; CI, confidence interval.

Model 1 was a crude model.

Model 2 for cotinine analysis adjusted for age, sex, race, PIR, smoking, hypertension, DM, dyslipidemia, depression, OSA, CRP, total energy intake, work status, work shift, and VD collection season.

### Potential regulating effect of vitamin D on the association between cotinine and insomnia

**Figure 2:**
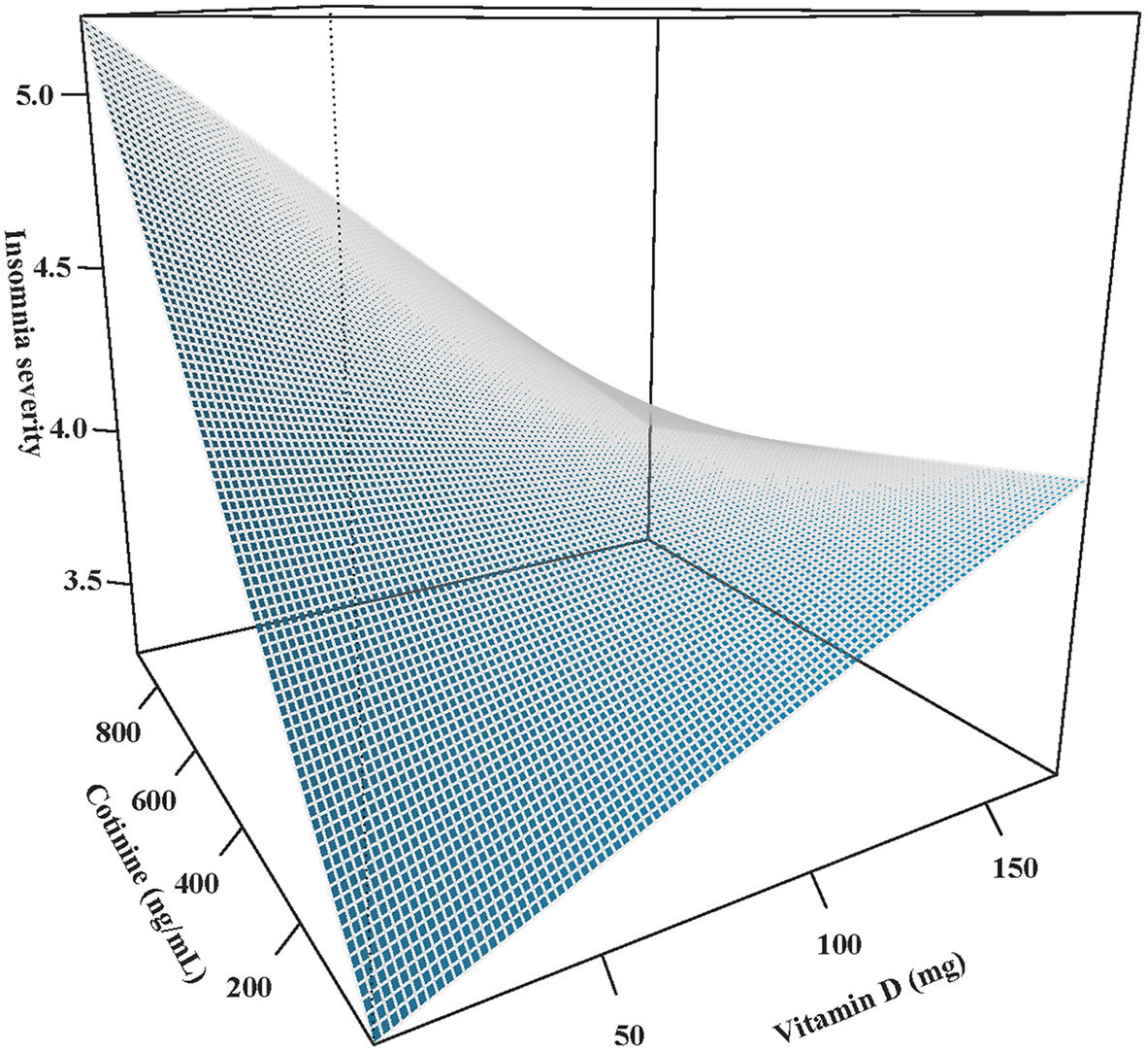
Effect of VD on the association between cotinine and insomnia.

Furthermore, we assessed the relationship between cotinine and insomnia at different serum VD levels (Table 3). In people with a serum VD level of <75 nmol/L, a higher serum cotinine level was associated with higher odds of insomnia [OR = 1.52, 95% CI: (1.17, 1.98)]. Interestingly, when serum VD concentrations were increased, this association became non-significant (*P* = 0.088). In addition, Figure 2 shows a surface diagram illustrating the role of VD in the association between cotinine and insomnia. Similarly, the severity of insomnia associated with serum cotinine concentration tended to decrease with the elevation of serum VD concentration, suggesting that a higher level of serum VD may have a potential regulating effect on tobacco exposure-associated insomnia.

**Table 3:** Relationship between serum cotinine level and insomnia in different serum VD levels.

**Serum VD levels, nmol/L**	**Serum cotinine levels, ng/mL**	**Model 1**		**Model 2**	
	**OR (95% CI)**	***P***	**OR (95% CI)**	***P***
< 75	< 0.05	Ref			
	0.05–2.99	1.01 (0.83–1.23)	0.942	1.03 (0.83–1.27)	0.821
	≥3.00	1.55 (1.22–1.98)	0.001	1.52 (1.17–1.98)	0.011
≥75	< 0.05	Ref			
	0.05–2.99	1.13 (0.72–1.75)	0.604	1.15 (0.74–1.80)	0.545
	≥3.00	1.38 (1.00–1.91)	0.063	1.75 (1.00–3.07)	0.088

VD, vitamin D; OR, odds ratio; CI, confidence interval.

Model 1 was a crude model.

Model 2 adjusted for age, sex, race, PIR, smoking, hypertension, DM, dyslipidemia, depression, OSA, CRP, total energy intake, work status, work shift, and VD collection season.

### Role of vitamin D in the association between cotinine and insomnia in subgroups of smoking status

We additionally explored the effect of serum VD level on the association between cotinine and insomnia in persons smoking or not (Table 4). After adjusting for covariates, the potential regulating effect of VD on the association between serum cotinine level and insomnia was found in participants who did not smoke. However, due to the limitation of the sample size, the odds of insomnia in the smoking subgroup could not be calculated, and the sample size of each subgroup is shown in Supplementary Table 2.

**Table 4:** Effect of serum VD levels on the relationship between serum cotinine level and insomnia in smoking subgroups.

**Serum VD levels, nmol/L**	**Serum cotinine levels, ng/mL**	**Non-smoking**		**Smoking**	
	**OR (95% CI)**	***P***	**OR (95% CI)**	***P***
< 75	< 0.05	Ref		Ref	
	0.05–2.99	1.06 (0.86–1.31)	0.588	0.36 (0.08–1.69)	0.221
	≥3.00	1.62 (1.23–2.12)	0.005	0.77 (0.20–2.94)	0.715
≥75	< 0.05	Ref			
	0.05–2.99	1.16 (0.74–1.82)	0.528		
	≥3.00	1.53 (0.86–2.70)	0.180		

VD, vitamin D; OR, odds ratio; CI, confidence interval.

Adjusted for age, sex, race, PIR, hypertension, DM, dyslipidemia, depression, OSA, CRP, total energy intake, work status, work shift, and VD collection season.

## Discussion

The current study found that adults with elevated serum cotinine levels seemed to have higher odds of insomnia, and a higher serum VD level may play a regulative role in this relationship. Moreover, the potential regulating effect of VD on the association between cotinine and insomnia was also found in those who were not smoking.

The role of tobacco smoke exposure in insomnia has been studied widely in recent years. A systematic review and meta-analysis of cohort studies investigated the impact of smoking on the incidence of insomnia, and the results showed that smoking could significantly increase the incidence of insomnia (26). Another systematic review and meta-analysis found an association between second-hand smoke exposure, short sleep duration, and poor sleep quality (7). Ph et al. (27) also provided evidence that smoking was related to increased insomnia severity and shorter sleep duration. In this study, we based our findings on the NHANES database and found a significant association between high serum cotinine concentration and high odds of insomnia in a representative population in the United States. Smoking might be an independent risk factor for insomnia, as suggested by several cross-sectional studies and epidemiological investigations (28–30). To date, nicotine, the primary addictive component of cigarettes or tobacco, is the most concerning. However, there is still no satisfactory explanation for the effects of nicotine on the human brain based on existing studies. Nicotine can affect the central release of dopamine, norepinephrine, serotonin, and acetylcholine, then enhance attention and maintain a certain level of arousal, which have been implicated in the regulation of wakefulness (31). Sleep parameter analysis (polysomnography, PSG) has detected the regulation and change in nicotine for the normal neurotransmitter and disturbances in sleep architecture both in the early and later stages of sleep (32, 33). Nevertheless, clinical practice guidelines indicate that insomnia is primarily diagnosed by history and that PSG is not indicated in the routine evaluation of insomnia (34). In our study, we diagnosed insomnia according to the NHANES questionnaires. In addition, psychosocial and physiological factors, such as depression, obesity, adverse life events, and work stress, may also account for the association between tobacco smoke exposure and insomnia (35–38). We evaluated the depression status of the study population and found that only 13.50% of them had depression. Since it was related to insomnia and was significantly different between the non-insomnia group and the insomnia group, we adjusted it in the multivariate models to make the results more robust. Future studies are needed to investigate this association and reveal the biochemical relationship between nicotine and insomnia.

Up to now, few studies have discussed the association between VD and insomnia. Similar to our findings, Soysal et al. (39) investigated older adults (aged 65 years or older) and found there was no significant difference between insomnia severity status and serum VD level. However, the MrOS Sleep Study by Massa et al. (40) showed that in older community-dwelling men aged ≥68 years old, low levels of total serum VD were associated with poorer sleep, including short sleep duration and lower sleep efficiency. Furthermore, a study among patients with chronic insomnia found that they had significantly lower VD concentrations compared with healthy controls (13). In addition, some influencing factors, such as age, shift work, and depression, have been recognized to play a role in the relationship between VD and insomnia. Patel et al. (41) considered that older adults were at higher risk for the medical and psychiatric effects of insomnia, and the factors contributing to late-life insomnia included demographic, psychosocial, biological, and behavioral factors. Park et al. (42) found that shift workers have more depressive symptoms, poorer sleep quality, and significantly lower VD levels than daytime workers. Although VD did not have any direct influence on sleep quality, the association between shift work and depression was mediated by sleep quality and VD (42). In the current study, we compared the work status and work shift between patients with insomnia and those without insomnia and adjusted these covariates in multivariate models. The mechanisms by which VD could affect sleep are not yet clear. Animal experiments have found nuclear concentrations of the VD hormone-target neurons in specific areas of the brain and spinal cord, which are thought to play a role in sleep (43, 44). A study of immunohistochemical investigations with antibodies to VD receptor proteins also found evidence for target neurons in the same regions of the brainstem and hypothalamus (12). The presence of VD target neurons in these regions of the brainstem that affect sleep suggests that VD may mediate an individual's sleep and further influence insomnia. Among our study population, VD was collected mostly from May 1 to October 31, and in the morning. Sunlight, diet, and supplements are all sources of VD for humans (45). It indicated that the participants may have relatively regular serum VD concentrations in this study. However, because the optimal 25-OH-D levels are still debated, and the effect of circadian rhythm on serum VD concentration is unclear, the relationship between VD and insomnia requires further exploration (46).

To the best of our knowledge, the current study first explored the role of VD in the association between tobacco smoke exposure and insomnia. Previous studies have reported a combined effect between VD and tobacco exposure on a variety of diseases, but insomnia was not included. Wu et al. (24) performed a cross-sectional study among adults from the NHANES in 2001–2016 to determine the relationship between VD levels and hypertension and the effect of tobacco smoke exposure levels on this relationship. Their results suggested that the increased risk of hypertension could be partly attributed to low VD levels induced by tobacco smoke exposure. Another study found that lower VD and smoking after clinical onset predicted worse long-term cognitive function and neuronal integrity in patients with multiple sclerosis (47). Our results indicated there may be an alleviating effect of high serum VD levels on associations with tobacco smoke-related insomnia. Nevertheless, the underlying mechanisms of the alleviating effect of VD on the association between tobacco smoke exposure and insomnia were not revealed. A meta-analysis compared circulating VD levels between smokers and non-smokers and found that smokers were likely to have lower circulating VD levels (47). Smoking has been reported to affect VD metabolism in many ways, including VD intake, synthesis, hydroxylation, and catabolism (48). A possible explanation for the alleviating effect of higher serum VD concentrations on insomnia related to smoking may be the role in affecting biological mechanisms linked between tobacco smoke exposure and depression, including monoamine imbalance, inflammation, altered stress response, oxidative stress, and dysfunction of brain-derived neurotrophic factor, which may further influence insomnia (20). Based on the use of hypnotics or other sleep therapy, dietary intake or supplements of VD are considered to be a complementary therapy with fewer side effects in clinical practice. Nevertheless, given the retrospective nature of this study, this potential regulating effect of VD needs further exploration in both basic and prospective cohort studies that provide more evidence for the application of nutrient supplements.

This study first explored the regulating effect of serum VD levels on the association between tobacco smoke exposure and insomnia, which may provide some idea for the early identification and prevention of high-risk populations that are exposed to insomnia. A wide range of covariates were controlled in the analyses, such as smoking, depression, OSA, work status, work shift, and VD collection season, and sensitivity analysis was performed to verify the robustness of our results. However, there are still some limitations to this study. As an observational study (cross-sectional study), we could not conclude the causal relationships between VD, cotinine, and insomnia or the exact regulating effect of VD in the association between cotinine and insomnia. In the NHANES, the diagnosis of insomnia was self-reported, which may cause recall biases. However, clinical practice guidelines indicate that insomnia is primarily diagnosed based on history and that PSG is not used for the routine evaluation of insomnia. Similarly, the dietary intake information was collected using two 24-h dietary recalls, which may cause recall biases. In addition, further prospective studies are needed to explore the causal relationship between VD and insomnia, as well as the potential regulative role of VD in the association between tobacco smoke exposure and insomnia.

## Conclusion

VD may play a potential regulative role in the relationship between tobacco smoke exposure and insomnia, which may provide some ideas for the early identification and prevention of high-risk populations for insomnia. However, further studies are needed to clarify the causal associations between VD, smoking, and insomnia.

## Data availability statement

Publicly available datasets were analyzed in this study. This data can be found at: NHANES database, https://wwwn.cdc.gov/nchs/nhanes/.

## Ethics statement

The requirement of ethical approval was waived by First Affiliated Hospital, Hebei University of Chinese Medicine for the studies involving humans because the study involved the analysis of existing datasets. The studies were conducted in accordance with the local legislation and institutional requirements.

## Author contributions

TG: Conceptualization, Data curation, Formal analysis, Project administration, Writing – original draft, Writing – review & editing. MH: Data curation, Formal analysis, Writing – review & editing, Conceptualization, Project administration, Writing – original draft. QW: Data curation, Formal analysis, Investigation, Software, Writing – review & editing. DL: Data curation, Formal analysis, Investigation, Software, Writing – review & editing. FC: Data curation, Formal analysis, Investigation, Software, Writing – review & editing. YX: Data curation, Formal analysis, Investigation, Software, Writing – review & editing. JM: Conceptualization, Supervision, Writing – original draft, Writing – review & editing.
