# Corrigendum: The roles of serum vitamin D and tobacco smoke exposure in insomnia: a cross-sectional study of adults in the United States

**DOI:** 10.3389/fnut.2025.1632721

**Published:** 2025-06-03

**Authors:** Tianci Gao, Mengxing Hou, Qianfei Wang, Dong Liu, Fenqiao Chen, Yueyi Xing, Jianqiang Mei

**Affiliations:** ^1^Graduate School, Hebei University of Chinese Medicine, Shijiazhuang, Hebei, China; ^2^Department of Emergency, First Affiliated Hospital, Hebei University of Chinese Medicine, Shijiazhuang, Hebei, China; ^3^Department of Traditional Chinese Medicine, Hebei General Hospital, Shijiazhuang, Hebei, China; ^4^School of Basic Medicine, Hebei University of Chinese Medicine, Shijiazhuang, Hebei, China

**Keywords:** cotinine, vitamin D, insomnia, regulating effect, NHANES

In the published article, there was an error in the **Funding** statement. The incorrect statement read as “This study was supported by Government-Funded Clinical Talents project of Hebei Province Finance Department (grant number: 13000022P00DE34100247). The correct statement should have been written as “This study was supported by Hebei Provincial Government Funded Training Program for Excellent Talents in Clinical Medicine (No. 13000022P00DE34100247); Graduate Student Innovation Grant Established Programs (No. XCXZZBS2024015).”

