# Supplementary material for: The roles of serum vitamin D and tobacco smoke exposure in insomnia: a cross-sectional study of adults in the United States
Source: Front Nutr. 2023 Dec 18;10:1285494. doi: 10.3389/fnut.2023.1285494 (PMC10759233; doi:10.3389/fnut.2023.1285494)
Supplement: Supplementary file 1 [file Data_Sheet_1.docx]

**Table S1 Characteristics of participants before and after the deletion of missing variables**

| Variables | After deletion (n=6312) | Before deletion (n=7173) | Statistics | *P* |
| --- | --- | --- | --- | --- |
| Age, years, Mean ± SE | 47.00 (33.00, 62.00) | 47.00 (34.00, 63.00) | Z=-1.014 | 0.311 |
| Gender, n (%) |  |  | χ2=0.207 | 0.649 |
| Male | 3278 (51.93) | 3697 (51.54) |  |  |
| Female | 3034 (48.07) | 3476 (48.46) |  |  |
| Race, n (%) |  |  | χ2=3.362 | 0.499 |
| Mexican American | 1234 (19.55) | 1447 (20.17) |  |  |
| Other Hispanic | 454 (7.19) | 548 (7.64) |  |  |
| Non-Hispanic White | 3033 (48.05) | 3345 (46.63) |  |  |
| Non-Hispanic Black | 1363 (21.59) | 1560 (21.75) |  |  |
| Other races | 228 (3.61) | 273 (3.81) |  |  |
| Education level, n (%) |  |  | χ2=7.300 | 0.121 |
| Less than 9th grade | 728 (11.53) | 928 (12.95) |  |  |
| 9-11th grade | 1043 (16.52) | 1188 (16.57) |  |  |
| High school grad/GED or equivalent | 1515 (24.00) | 1723 (24.04) |  |  |
| Some college or AA degree | 1745 (27.65) | 1940 (27.06) |  |  |
| College graduate or above | 1281 (20.29) | 1389 (19.38) |  |  |
| PIR, n (%) |  |  | χ2=0.507 | 0.476 |
| ≤1 | 1159 (18.36) | 1272 (18.85) |  |  |
| >1 | 5153 (81.64) | 5477 (81.15) |  |  |
| Work status, n (%) |  |  | χ2=3.281 | 0.070 |
| Working at a job or business | 3958 (62.71) | 4389 (61.19) |  |  |
| Not working at a job or business | 2354 (37.29) | 2784 (38.81) |  |  |
| Work shift, n (%) |  |  | χ2=3.674 | 0.452 |
| A regular daytime schedule | 2958 (46.86) | 3259 (45.43) |  |  |
| A regular evening/night shift | 357 (5.66) | 412 (5.74) |  |  |
| A rotating shift | 297 (4.71) | 334 (4.66) |  |  |
| Another schedule | 345 (5.47) | 383 (5.34) |  |  |
| Unknown | 2355 (37.31) | 2785 (38.83) |  |  |
| BMI, kg/m^2^, n (%) |  |  | χ2=0.175 | 0.675 |
| ≤25 | 1827 (28.94) | 2077 (29.27) |  |  |
| >25 | 4485 (71.06) | 5018 (70.73) |  |  |
| Physical activity, MET · min / week, n (%) |  |  | χ2=2.556 | 0.279 |
| ≤450 | 899 (14.24) | 1015 (14.15) |  |  |
| >450 | 3425 (54.26) | 3808 (53.09) |  |  |
| Unknown | 1988 (31.50) | 2350 (32.76) |  |  |
| Drinking, n (%) |  |  | χ2=0.175 | 0.676 |
| No | 1829 (28.98) | 2000 (29.31) |  |  |
| Yes | 4483 (71.02) | 4824 (70.69) |  |  |
| Smoking, n (%) |  |  | χ2=0.043 | 0.836 |
| No | 4877 (77.27) | 5553 (77.42) |  |  |
| Yes | 1435 (22.73) | 1620 (22.58) |  |  |
| Hypertension, n (%) |  |  | χ2=0.004 | 0.951 |
| No | 2949 (46.72) | 3347 (46.67) |  |  |
| Yes | 3363 (53.28) | 3825 (53.33) |  |  |
| DM, n (%) |  |  | χ2=0.640 | 0.424 |
| No | 5390 (85.39) | 6090 (84.90) |  |  |
| Yes | 922 (14.61) | 1083 (15.10) |  |  |
| Dyslipidemia, n (%) |  |  | χ2=0.037 | 0.848 |
| No | 1660 (26.30) | 1876 (26.15) |  |  |
| Yes | 4652 (73.70) | 5297 (73.85) |  |  |
| Depression, n (%) |  |  | χ2=0.725 | 0.394 |
| No | 5504 (87.20) | 5885 (86.70) |  |  |
| Yes | 808 (12.80) | 903 (13.30) |  |  |
| OSA, n (%) |  |  | χ2=0.264 | 0.607 |
| No | 3117 (49.38) | 3574 (49.83) |  |  |
| Yes | 3195 (50.62) | 3599 (50.17) |  |  |
| CRP, mg/dL, Mean ± SE | 0.20 (0.08, 0.47) | 0.20 (0.08, 0.46) | Z=0.271 | 0.786 |
| Total energy intake, Kcal, Mean ± SE | 1950.00 (1441.00, 2645.50) | 1936.00 (1423.00, 2624.00) | Z=1.097 | 0.273 |
| Caffeine intake, mg, Mean ± SE | 101.00 (16.00, 225.00) | 101.00 (15.00, 219.00) | Z=1.008 | 0.314 |
| HEI-2015, Mean ± SE | 49.91 ± 13.19 | 50.10 ± 13.23 | t=-0.87 | 0.384 |
| VD collection season, n (%) |  |  | χ2=0.079 | 0.779 |
| November 1 through April 30 | 2879 (45.61) | 3289 (45.85) |  |  |
| May 1 through October 31 | 3433 (54.39) | 3884 (54.15) |  |  |
| Blood collection time, n (%) |  |  | χ2=0.005 | 0.998 |
| Morning | 3051 (48.34) | 3468 (48.35) |  |  |
| Afternoon | 2297 (36.39) | 2607 (36.34) |  |  |
| Evening | 964 (15.27) | 1098 (15.31) |  |  |
| Cotinine, n (%) |  |  | χ2=0.084 | 0.959 |
| <0.05 | 2754 (43.63) | 3128 (43.61) |  |  |
| 0.05-2.99 | 1743 (27.61) | 1995 (27.81) |  |  |
| ≥3.00 | 1815 (28.75) | 2050 (28.58) |  |  |
| VD, nmol/L, n (%) |  |  | χ2=0.309 | 0.578 |
| <75 | 5079 (80.47) | 5799 (80.84) |  |  |
| ≥75 | 1233 (19.53) | 1374 (19.16) |  |  |

M: mean, SE: standard error, PIR: poverty-income ratio, BMI: body mass index, MET: metabolic equivalent, DM: diabetes mellitus, VD: vitamin D, OSA: obstructive sleep apnea, CRP: C-reactive protein, HEI: Healthy Eating Index.

**Table S2 Sample size in subgroup of different smoking status**

| Serum VD levels  nmol/L | Serum cotinine levels  ng/mL | Non-smoking, n (%) | Smoking, n (%) |
| --- | --- | --- | --- |
| <75 | <0.05 | 2190 (55.50) | 10 (1.08) |
|  | 0.05-2.99 | 1399 (35.57) | 47 (3.66) |
|  | ≥3.00 | 369 (8.93) | 1815 (29.59) |
| ≥75 | <0.05 | 552 (59.54) | 2 (1.23) |
|  | 0.05-2.99 | 287 (30.69) | 10 (3.22) |
|  | ≥3.00 | 80 (9.77) | 302 (95.55) |

VD: vitamin D.
